# Recognition of the Driving Style in Vehicle Drivers

**DOI:** 10.3390/s20092597

**Published:** 2020-05-02

**Authors:** Jorge Cordero, Jose Aguilar, Kristell Aguilar, Danilo Chávez, Eduard Puerto

**Affiliations:** 1 Departamento de Ciencias e la Computación y Electrónica, Universidad Técnica Particular de Loja, Loja 110107, Ecuador; 2 Grupo de Investigación, Desarrollo e Innovación en TIC, Universidad EAFIT, Medellín 050021, Colombia; 3 Centro de Microcomputación y Sistemas Distribuidos, Universidad de Los Andes, Mérida 5101, Venezuela; kristell153@gmail.com; 4 Escuela Politécnica Nacional, Quito 170525, Ecuador; danilo.chavez@epn.edu.ec; 5 Grupo de Investigación en Inteligencia Artificial, Universidad Francisco de Paula Santander, Cúcuta 540001, Colombia; eduardpuerto@ufps.edu.co

**Keywords:** pattern recognition, driving style, intelligent techniques, advanced driver-assistance systems

## Abstract

This paper presents three different approaches to recognize driving style based on a hierarchical-model. Specifically, it proposes a hierarchical model for the recognition of the driving style for advanced driver-assistance systems (ADAS) for vehicles. This hierarchical model for the recognition of the style of the car driving considers three aspects: the driver emotions, the driver state, and finally, the driving style itself. In this way, the proposed hierarchical pattern is composed of three levels of descriptors/features, one to recognize the emotional states, another to recognize the driver state, and the last one to recognize the driving style. Each level has a set of descriptors, which can be sensed in a real context. Finally, the paper presents three driving style recognition algorithms based on different paradigms. One is based on fuzzy logic, another is based on chronicles (a temporal logic paradigm), and the last is based on an algorithm that uses the idea of the recognition process of the neocortex, called Ar2p (Algoritmo Recursivo de Reconocimiento de Patrones, for its acronym in Spanish). In the paper, these approaches are compared using real datasets, using different metrics of interest in the context of the Internet of the Things, in order to determine their capabilities of reasoning, adaptation, and the communication of information. In general, the initial results are encouraging, specifically in the cases of chronicles and Ar2p, which give the best results.

## 1. Introduction

Currently, there is interest in the development of Advanced Driver-Assistance Systems (ADAS) for vehicles [1,2,3,4]. A context-aware ADAS aims to assist drivers according to the current situation. In this context, the relationships between the car drivers and the ADAS is an important aspect to consider, and more particularly, how the ADAS can be adapted to the characteristics of each car driver [1,2,4,5]. From the above, the following questions arise: How should mechanisms of adaptation be incorporated for each driver in the ADAS? What factors should be considered to recognize a driving style? How are these factors related?

Particularly, to allow the adaptation of an ADAS to each driver, it is necessary to recognize the main human factors that the driver can have in a given moment, which have a great influence on the driving style [6,7,8,9]. Also, with respect to the driving style, there are other factors that influence it, such as the emotions and states of the driver, which also are necessary to recognize [1,3,10]. However, to recognize any of the three, it is necessary the definition of the set of descriptors/features that determine them. In this sense, this paper analyzes these factors in such a way as to establish the set of descriptors that could be used to describe them in real contexts. The descriptors determine those variables that should be observed by the ADAS, to identify the different states, styles, or emotions that can have a driver. Based on the descriptors, it is possible to define specific patterns of the states, styles, or emotions of a driver.

Initially, the paper analyzes how to measure the descriptors of the hierarchical model, which forces to assume a perceptual multimodal approach. Once the different style, state, and emotion patterns of a driver are defined, the paper analyzes the techniques that can model those patterns in the context of an ADAS. These techniques should not only recognize each possible emotion, state or style, but they should also be able to infer possible causes of the same, to help the ADAS to define the possible actions to be executed. The main contributions presented in this article are:

(i) The combination of the hierarchical models of driving styles proposed in [6] with the emotional recognition approach proposed in [10] for a new hierarchical multimodal model (sound, vision, etc.) for driving styles recognition.
(ii) The implementation of three recognition algorithms based on different paradigms: Chronicles, Ar2p algorithm, and fuzzy logic. The first paradigm is based on a set of events linked by a set of temporal constraints, the second paradigm is based on the operation of the neocortex, and the third paradigm is based on the fuzzy theory. These paradigms were selected due to their capabilities to manage partial and ambiguous information, which can occur in an ADAS.
(iii) Finally, an exhaustive comparison using relevant criteria in an internet of things (IoT) context, and for ADAS and cruise adaptive control (ACC) [11] applications. These criteria are of three types, to analyze the three techniques from different points of view, in order to determine their capabilities in areas of interest for an ADAS or ACC [2,5,12]. The first capability is the reasoning, in order to deduce the possible states, styles, and emotions, as their possible causes. The next capability is the learning, in order to learn the patterns of each driver, understanding that it is specific for each one. Finally, the other capability is linked to the recognized information that is transmitted, a vital aspect in the context of the Internet of the Things (IoT), where the information must travel fast between the objects of the environment, in this case, between vehicles (their ADAS and ACC) and/or drivers.

This paper is organized as follows: Section 2 presents a literature review. Then, Section 3 analyzes the hierarchical pattern of the driving style proposed in [6], to modify it with descriptors that can be sensed in real contexts. Section 4 presents three recognition approaches based on different intelligent techniques. The next section presents a comparison of the techniques, defining a set of metrics to determine their capabilities of reasoning, learning, and communication. For that, various scenarios of driving situations to test these capabilities, using real data, are defined. Finally, the conclusions are presented.

## 2. State of the Art

Lin et al. propose an adaptive ADAS that considers the internal characteristics of each human being, like fatigue, inattention, and specifically, the driving type [7]. The authors propose a set of driver characteristics and review the key technologies to determine the behavioral characteristics of the driver, such as the data acquisition techniques, or the classification and identification methods of the driver behavior. The authors of [5] propose the design of the control strategy for an ACC, according to the driving types. In this work, they consider that a safe driver wants ACC to work before, while the aggressive driver wants ACC to work later. They include these driver features in the design of the control algorithm, in order to make the ACC system suited to the car drivers. In [13], the authors propose a control system for an ADAS. The controller is designed to mitigate the negative effects produced by possible visual distractions of the driver. In addition, the paper evaluates the user’s visual distraction and its effect on two aspects: with respect to the path and with respect to the obstacles. They associate two-time delays around these effects and propose a control scheme that considers distraction in the design. Slawiñski et al. [14] propose a driver alert system based on the feedback of vibrotactile stimuli of force, to prevent traffic accidents. The system can easily be mounted on any vehicle, and it uses wireless communication with other vehicles to warn the driver of a possibly dangerous situation in the next few seconds. The model is focused on human factors, and the system is tested on an open-source 3D racing simulator.

There are several works about emotions in a car because it is a great challenge to personalize the ADAS to each driver. For example, Guoying et al. [15] propose a pattern recognition approach to identify the driver behavior. Their approach is divided into three phases: the parameter extraction, the clustering process, and the identification model building. They use a K-means algorithm and a Gaussian mixture model for the clustering process. Based on the clusters, they build two identification models of the driver behavior, using an artificial neural network and a support vector machine, for the recognition of the driver’s driving habits. The aim of [8] is to explore the effects of the emotions on the driving performance and workload. They analyze the impact of the affective states of the driver. For that, they use a vehicle simulator under three different road conditions, with one of the following induced affective states: anger, fear, happiness, or neutral. They measure the subjective judgment, the risk perception, and the safety level. They suggest that it is necessary to take into account the emotions, in order to construct a generic driving behavior model. For example, Anger has adverse effects on the personal safety level and degrades the driving performance. In [9], the maladjusted driving is analyzed, such as aggressive driving, and its relationship with the traffic accidents. They propose that the effects of the emotions in the traffic are divided into two distinct classes: personal factors and driving situations. They carry out simulations in four traffic situations where each situation has critical elements (e.g., slow car, an obstacle in the street). Their results indicate that the anger leads to a stronger acceleration and higher speeds, and that anxiety has a similar behavior, but with weaker effects.

In [3], the authors investigate the potential to identify individuals using sensor data snippets about their driving behavior. Their results indicate that drivers are distinguishable using only in-car sensors. In particular, they can differentiate the drivers with 100% accuracy when the training is with all the available sensors. When more training data is available, it is possible to reach a very good identification using only a single sensor (e.g., the brake pedal). The paper [10] proposes a recognition model of the emotions, using chronicles. In this work, they present only the model of recognition based on chronicles, which is not implemented and tested in real situations. The authors of [16] propose a pattern recognition approach to characterize a driver’s behavior. Their goals are to shorten the recognition time and improve the recognition of driving styles, using a k-means clustering-based support vector machine (kMC-SVM) method, for classifying drivers into two types: aggressive and moderate. They use the vehicle speed and the throttle opening as the parameters to characterize the driving styles. In [1], the authors present a revision of the works in emotion recognition, focusing on those influencing the driver’s performance. They analyze the influence of the emotions in the driving behavior, based on the traffic situations and the driver risk tolerance. The paper is focused on the definition of an alerting mechanism and of a driver state recognition mechanism, which includes the driver’s stress and fatigue. Lau et al. [17] investigated how positive and negative emotions affect the driving speed, the steering, and the hazard response times. Contrary to expectations, results revealed no significant effect of emotional valence on the speed and steering. Furthermore, there is an interaction between the valence and the hazard situation that reduces the braking time. These findings suggest arousal to have an important role in driving attention mechanisms. Kamaruddin et al. [18] study the speech recognition problem. They use the real-time recorded speech from drivers, in order to analyze the performance in a vehicular setting. They identify three basic emotions, namely angry, sad, and happy. They define the speech emotion profile, to explore its universality and diversity. Dörr et al. present a system for online driving style recognition [19]. They use fuzzy logic for identifying the current driving style, which can be adapted to nearly every car.

It is expected that the autonomous vehicles with the capability of driving without human supervision will be released to the market in this decade. One first work in this domain is [2], wherein the authors propose a learning approach that allows the user to demonstrate the desired driving style to the car. They define parameters, such as the acceleration profiles, the distances to other cars, the speed during lane changes, etc., to characterize a human driver’s style. They use a feature-based inverse reinforcement learning, to find the model parameters that fit the observed style. Once the model has been learned, it can be used by the vehicle to compute trajectories. In [20], the authors propose a deep learning solution for characterizing the driving styles using the Global Positioning System (GPS). They propose an approach that can extract high level and interpretable features, which describe complex driving patterns. The learned driving styles are validated using a real dataset.

There are several works about the emotions of the car driver [12,21,22,23,24,25], but in general, they propose simple models, they study only the emotions, they do not evaluate the quality of the recognition approach, or the capabilities of the recognition approach, in real contexts. These aspects are covered in this paper. Specifically, the previous works define very simple patterns to determine the behavior of the driver (in some cases, they consider only the emotions, and in general, they do not consider the driver state), and they do not analyze which descriptors can be measured in an ADAS real context. Also, there are several works about driving style recognition techniques that are not analyzed from the point of view of IoT, with metrics specifically linked to the learning, reasoning, and communication. This paper conjugates all these aspects, to make an exhaustive analysis of the problem of recognition of driving styles for ADAS.

## 3. Formal Definition of the Pattern of Driving Style

In general, the driving style has been defined in the literature as the attitude, orientation and way of thinking of the daily driving [2,16,19,20,26,27]. One of the main aspects for the recognition of the driving style is the definition of the patterns with their descriptors/features. Based on the patterns, it is possible to define recognition algorithms and test their capabilities. In this way, the first step is to analyze the definition of the patterns.

**Figure 1:**
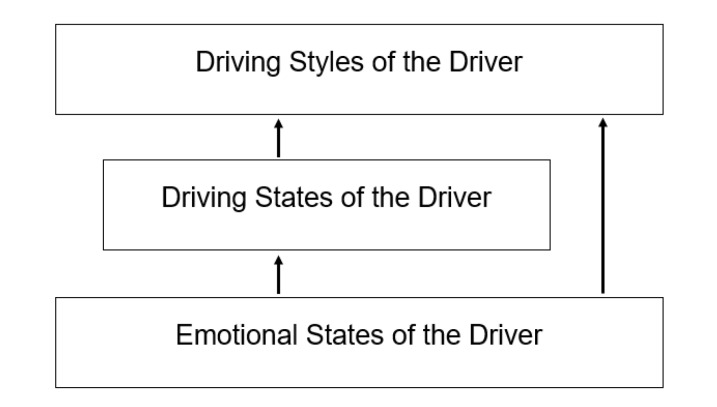
Hierarchical pattern of driving styles.

In [6] different types of descriptors are presented to offer a good description of the context, but some of them cannot be obtained in a real context. For this reason, it is necessary to determine the descriptors that can be sensed in an ADAS real context. In this work is proposed a set of characteristics (descriptors) to describe each one of the aspects of the driver, which can be measured in a real context. In this way, the theoretical pattern defined in [6] is modified. Remember that the factors that must be considered of a driver are the driving styles, the driver states, and the driver emotions. In that sense, it is necessary for each of them, the determination of a group of characteristics that define them. Particularly, one of the characteristics of the driver’s state is the emotions, so that to recognize the state it is necessary previously the recognition of the emotion. Equally, in order to recognize the driving style, it is required the recognition of both the state and the emotion of the driver. Thus, a hierarchical relationship is established between the patterns of each factor to be considered, to describe a driver (see Figure 1).

Aguilar et al. [6] describe a preliminary form of this hierarchical model. In general, the pattern is defined by a set of descriptors by level, which defines the characteristics of each factor to be recognized (emotion, state, and style). In this way, the hierarchical pattern defined in [6] is composed of three levels, which in this paper is modified according to the descriptors that can be sensed in an ADAS real context:

First level: Pattern of the driving style. It aims model how the driver drives. Classically, the driving style is defined in the literature as aggressive, ecological, urban, and normal [4,9,22,23]. The proposed model allows for the recognition of the driving style considering the set of descriptors defined in Table 1.

Second level: Driver state. This level describes the state of the car driver. Normally, the state of a car driver is described in the literature as wakeful, stressed, lethargic, pleasant, fatigued, concentrated, calm, impatient, boring, and falling asleep among others [4,9,22,23]. To detect the current state of the driver, the following descriptors are proposed, as shown in in Table 2.

Third level: Emotions of the Driver. This level describes the emotions of the driver. Particularly, the six basic emotions defined in the literature are [28]: happiness, sadness, fear, anger, disgust, and surprise. Each emotion is defined by the set of descriptors proposed in Table 3.

The main goal of the hierarchical pattern is to recognize the driving style, in order to be used by an ADAS. To recognize the driving style, it is necessary the descriptors defined in Table 1, which include the state and emotion of the driver. Equally, to recognize the state of a driver, the descriptors of Table 2 are used, and one of them, is the emotion. Thus, each level has a different set of descriptors, each of which are perceived in different ways (sound, vision, etc.). That is, ADAS requires a perception system composed of different types of sensors, in order to instance the descriptors of each level. That implies the use of a perceptual multi-modal approach, with, for example, sound sensors, but also, with mechanisms for the processing of the captured information. For example, an image processing system may be required to recognize the driver’s face, the body language, etc.

However, not all descriptors are captured in real-time, and not all descriptors have the same frequency of change, some of them are extracted from databases (e.g., personal information of the driver), others are directly captured from the vehicle using sensors in the car. In this way, our hierarchical model requires a perceptual multimodal system.

In our case, the current status of the descriptors determines the event at a given moment. For that, our approach uses the information from the different sensors in the perceptual multimodal system. In our case, each time that changes a descriptor means a new event. In this way, there are two types of events, one where only a descriptor changes its value, called a simple event, and another where several descriptors change their values, called complex event.

In this way, our model is very sensible, and according to the current values of the descriptors, then it determines the current emotion, the current state, and finally, the driving style of the driver. Examples of the possible driving styles that could recognize our model are shown in Table 4. For instance, according to the value of the descriptors of this pattern, our model can detect an aggressive driving style. In this example, if the state of the driver is stressed, their emotion is anger, the road is normal, it is raining, and the road has potholes. In the same way, according to the values of the descriptors in a given moment, other driving styles would be recognized, even the same style but with other values in the descriptors. The same happens in the case of recognition of emotions and driving states.

In general, in Table 4, each driving style is associated with different descriptor values [21,22,26,27,29]. For example, the aggressive driving style is associated with the stress (state) and the anger (emotion). However, additionally, the rainy weather and the condition of the pothole on the road define this driving style. On the other hand, the ecological driving style is associated with a relaxed state and the happiness (emotion). Finally, a driving style can have several patterns (set of descriptor values), and it is possible to add new driving styles using these descriptors. In Table 5, the descriptors have been defined like codes, in order to simplify and encode the information represented by them, in such a way to semantically enrich them. Table 5 presents an example of this information encoded in the descriptors, for the case of the emotions.

In this example, the happiness of the driver can be recognized if the driver’s voice is soft and low, the facial expression is neutral, the driver uses the horn normally, the brake light is turned off, the speed is 100 Km/h, the tires are new, the driver looks on the road, and the hands are on the steering wheel, among other things. In this way, the hierarchical model gives a detailed explanation of the conditions that determine an emotion. It is similar, in the case of the state of the driver and the driving style.

Table 1, Table 2 and Table 3 define the set of descriptors for the recognition of a driving style. In a real context, some of the descriptors may not be captured or their capture may be intermittent due to problems with the sensors, or the instrumentation installed may not contemplate them due to their complexity to deploy them or because they are intrusive to the driver. In this sense, the methods for processing driving styles must always be able to handle these uncertain situations, with partial and ambiguous information. These aspects will be analyzed in the experimental part of this work.

## 4. Approaches for the Modeling of the Styles of Driving

In this section are explained the three paradigms used in this work, in order to recognize the driving styles. The first paradigm is based on chronicles, the second paradigm is based on the operation of the neocortex, and the third paradigm is based on the fuzzy logic. Specifically, the goal of this section is to show the capabilities of the different intelligent techniques to model our complex pattern of driving styles. These paradigms have been selected because they can process partial and ambiguous information. This is typical in an ADAS context, where not all information is available or is correct in a given moment, for several reasons, including communication faults, and sensor faults among other things [4,5].

### 4.1. Based on Chronicles

A chronicle can be defined as a set of events, linked by a set of temporal constraints [30,31]. Each chronicle is an event pattern with temporal relationships between them, and a set of chronicles describes the possible evolution of the studied system. In general, a chronicle model C is defined by a pair (S, T), where S is the set of events and T the temporal constraints between the events. An instance c of a chronicle model C is a set of event occurrences, which is consistent with the time constraints of C. To define a chronicle, normally two predicates are used: event and hold. An event expresses a change in an attribute, for example: Event (state (light): (on, off), t2). A hold specifies that an attribute holds a value during a time interval, for example: Hold (position (robot, home), (t2, t4)).

Now, it is described how can be modelled the hierarchical pattern using chronicles. Each chronicle is defined by a set of the descriptors defined in the previous section, which define the events and the temporal relationships to recognize it. In general, every emotion, state, or driving style of the driver will be modelled by a different chronicle. However, the same emotion, state, or driving style can be recognized by several chronicles. In this context of this application, a chronicle is defined by a set of events, described by the values that each descriptor takes from the hierarchical pattern at a given moment (sensed value) and its moment of occurrence, and the temporal relationship between those events (how much sooner or later those sensed values occur between them). In this way, it is possible to capture behaviors more complex than a simple yawn or abrupt maneuver to deduce that it may be happening. Specifically, our hierarchical model proposed in the previous section consists of three types of chronicles:

(i) The first type of chronicle represents the emotional patterns of the driver. It aims to describe the emotions of the driver. An example of a chronicle of the first type, to recognize the anger, is:

Chronicle Anger {
    event (V1, T1),
    event (S1, T2)
    event (P1, T3),
    event (F3, T4),
    event (B5, T5),
    event (H1, T6),
    T1 > T3,
    hold (V1, (4, 10)),
    HOLD (S1, (6, 20)),
When recognized {emit event(ED1)}}
		

According to this chronicle, the pattern of anger can be recognized when the voice event “Tone treble and volume high and speaking rate fast” (V1) arrives at time T1, and holds between 4 and 10 units of time; the speed event “High speed” (S1) occurs at the time T2, and holds between 6 and 20 units of time, the pressure event “Strong pressure on the steering wheel” (P1) appears at time T3 and it is less than T1, the facial event “Eyes and Eyebrows open, with curves and tight lips, and face wrinkles in the centre” (F3) occurs at time T4, the body event “Posture Flattened” (B5) occurs at time T5, and the heart event “Fast Heart rate” (H1) arrives at time T6.

(ii) The second type of chronicle represents the patterns of the driver state. It aims to describe the driver’s condition. An example of a chronicle of the second type, to recognize a stressed driver, is the following:

Chronicle Stressed {
    event (ED1, TED1), 
    event (DE2, T1), 
    event (TS2, T2), 
    event (S2, T3) 
    T3 < T2, 
    T3 < T1, 
    TED1 < T2, 
    TED1 < T1, 
    hold (S2, (2, 20))
When recognized {emit event(ST3)}} 
		

Where, ED1 is the event generated when the Anger emotion is recognized at the time TED1, and DE2, TS2 and S2 are events generated by the descriptors of the driver state.

(iii) The third type of chronicle represents the patterns of the driving styles. It aims to establish how the person drives. An example of a chronicle of the third type, to recognize an aggressive driver, is the following:

Chronicle Aggressive {
    event (ED1, TED1), 
    event (ST3, TST3), 
    event (R1, T4), 
    event (E2, T3) 
    TED1 > TST3, 
    T3 > T4,
    hold (ST3, (5, 15)),
When recognized {Report the driving style to the ADAS}}
		

Where, ST3 is the event generated when the Stressed state is recognized at the time TST3, and R1 and E2 are events generated by the descriptors of the driving style.

This is just a sample of the proposed chronicles used by an ADAS, where the chronicles of type 1 and 2 are composed of the primary events captured through different types of sensors (pressure sensor on the steering wheel, driver’s heart rate sensor, and speed sensor, among others). Chronicle type 3 is a mixture of the primary events and events recognized in the hierarchical system. Chronicles can consider ambiguous situations such as when an event does not occur what to do, or the variable temporal occurrence between events, among other things.

**Figure 2:**
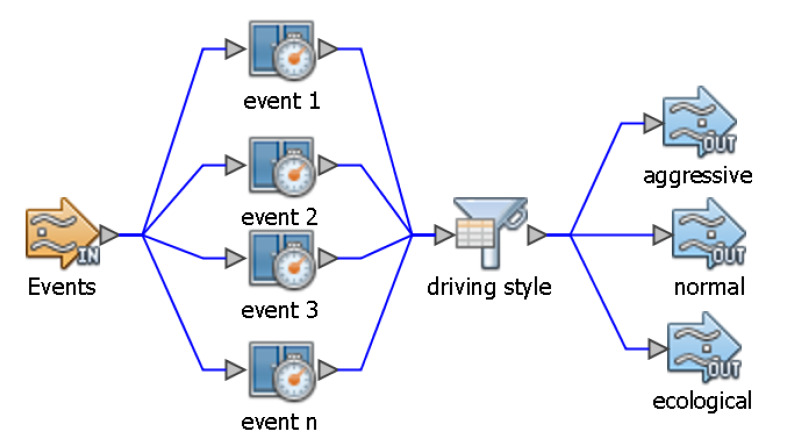
An example of the definition of a chronicle using the IEP component in OpenESB.

The implementation of the learned chronicles is carried out in a tool called OpenESB. OpenESB is a service-oriented middleware used to build service-oriented architecture (SOA) applications. It has a component, called intelligent event processor (IEP), which allows the processing of complex events, the base of the motor of inference for the recognition of chronicles. It uses the continuous query language (CQL) to describe the chronicles, which is a declarative language that performs continuous queries on an event flow. Figure 2 shows an example of a chronicle, using the IEP component.

The event component represents an input stream that collects a series of events. These events define the changes in the descriptors that define the chronicles. According to Figure 2, there are several events (event 1, event 2, event 3, and event n), which are events that occur in the descriptors that are part of the driving style pattern.

The representation of chronicles using the CQL language is the following. CQL is a query language, which is declarative, used to perform continuous queries on an event stream. Syntactically, CQL is very similar to the SELECT statement of the structured query language (SQL), but the execution of the queries is different of the queries of conventional databases in SQL, whose queries are executed on demand, until all requested data is completed. By contrast, in CQL, the queries are continuous streams of data, running indefinitely (infinite tuples of streams), or until the application that invokes ends. CQL has the same operators that SQL, like projection, selection, aggregation, joining, grouping, etc. It also has other operators that allow relating stream to relations. For more details about CQL, see [32]. Taking the chronicle model defined previously, which recognizes the patterns of anger, this chronicle can be expressed in CQL statements as follows:

Chronicle Anger {
    SELECT
    ISTREAM {
    event_level2 => ’EStressed’
    event_leve1 => ’EAggressive’
    ‘Anger Recognized’
    }
    FROM
    eventV1[T1]
    eventS1[T2]
    eventP1[T3]
    eventF3[T4]
    eventB5[T5]
    eventH1[T6]
    WHERE
    eventP1.time < eventV1.time
Where each event_i_ is a descriptor.
		


### 4.2. Based on Ar2p

**Figure 3:**
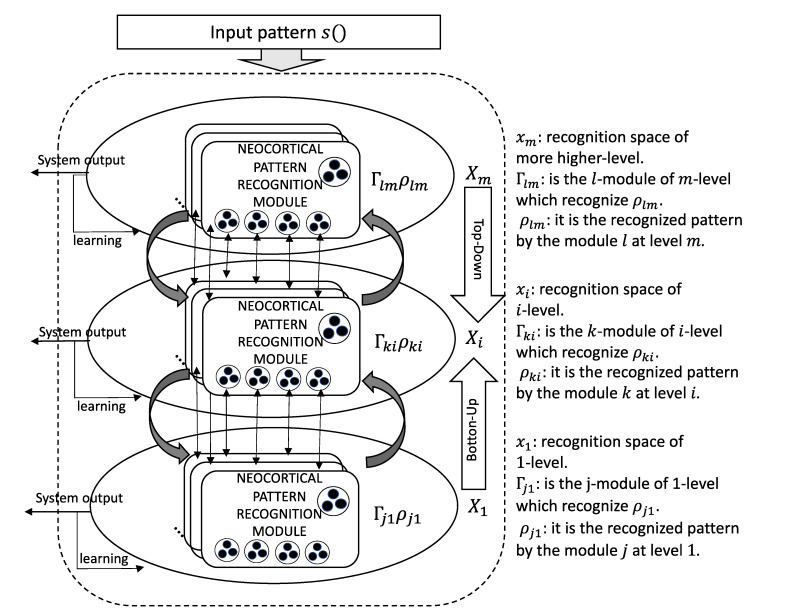
Recursive pattern matching model (Ar2p).

Ar2p is a model for pattern recognition, inspired by the pattern recognition theory of mind [33,34]. Ar2p is based on the neocortex pattern recognition process. The hierarchical recognition model in Figure 3 represents the recursive and iterative process of Ar2p. Each layer in the hierarchy is an interpretation space (ovals) identified as X_i_, from i = 1 to m. X_1_ is the level of recognition of the atomic patterns, and X_m_ is the level of recognition of the complex patterns (for example, a driving style). Each level is composed of Γ_ji_ recognition modules (for j = 1, 2, 3... # of modules at level *i*), where ρ_ji_ is the recognized pattern by the module j at level i.

The input pattern *s*() in Figure 3 represents the presence of a pattern to be recognized. For the top-down recognition case, the output signal of the higher-levels is the input signal at the lower-levels. Each recognition module Γ*_ji_* (which recognizes its corresponding pattern ρ_ji_) has a relationship of structural composition with the recognition modules of lower-levels, such that Γ_ji_ → Γ_lk_, where l defines the number of the recognition module at level k, and i > k. Relationship “→“ indicates that the module Γ_lk_ of X_k_ is contained or is part of the module Γ_ji_, which belongs to layer X_i_ of higher level. In other words, a Γ_j_ of X_i_ is composed of different Γ_l_ of X_k_ of lower level. There may be different versions of the same pattern (redundancy/robustness) represented by different Γ_ri_, from r = 1, 2, 3 ... until possible variations of the object in the real world. Each level i produces an output signal (recognition or learning) based on the responses of its modules. The output of each Γ_ji_ consists of a specific signal of recognition of its pattern ρ_ji_, which is transmitted through the dendrites to its higher levels. This signal contains information about the characteristics of the pattern that represents. This process is valid for both, top-down and bottom-up processes (for more details about these processes, see [33,34]). Such recognition is diffused through all the dendrites to which the recognition module is connected. When it is not recognized, then it sends a signal that maybe involves learning [34].

A pattern matching module is formally defined as a 3-tuple Γ = < *E*, *U*, *S_o_*>, where: *E* is an array composed of 2-tuple *E* = <S, C> (see Table 4); *S* = <Signal, State> is an array that represents the set of signals that conform to the pattern recognized by Γ and their respective states; *C* is an array that encodes information about the pattern, defined by a 3-tuple *C* = <D, V, P>, where D represents the descriptors of Γ, V is the domain vector for each D, and W it the weight (importance) of each D for the recognition of the pattern. Table 6 shows the structure of the recognition model.

On the other hand, in [33,34] have been defined two strategies for recognition of a pattern: the first strategy by key signals, and another strategy by partial recognition of signals. The first uses the weight of importance of the input signals identified as keys, and the second uses the partial or total presence of the signals.

**Theorem 1.** *Strategy by key signals. This recognition uses the descriptors (signals, sub-patterns) with greater weight of importance and the ΔU1 threshold for the recognition.*

**Definition 1.** *A key signal. A s_i_ signal in the Γ module is key if its importance weight has a value greater or equal than the average of all the signals in Γ. The formula is:*

∀*s_i_ ∈S(*Γ), if [W(*s_i_*) *≥* W_average *S(*Γ)]→Clave_Γ_(*s_i_*) (1)

**Definition 2.** *Recognition by key signals. A ρ pattern is recognized by key signals if:*

(2) $$\frac{\sum_{i=1\;\cap^{\text{​}}\;state\left(s_{i}=true\right)\cap^{\text{​}}s_{i}\;\in {\mathrm{Clave}}_{\Gamma }\;}^{n}\;W\left(s_{i}\right)}{\left|{\mathrm{Clave}}_{\Gamma }\right|}\;\geq \;∆U1\to \;S_{o}$$

If the average of the weights of the recognized key signals > Δ*U*1, then the pattern is recognized.

**Theorem 2.** *Strategy by partial mapping. This strategy consists of the validation that a signal number minimum present in Γ is superior to the ΔU2 threshold.*

(3) $$\frac{\sum_{i=1\;\cap^{}\;state\left(s_{i}=true\right)\;}^{n}\;W\left(s_{i}\right)}{n}\geq \;∆U2\;\to \;S_{o}$$

If the average of the active input signals in Γ > Δ*U*2, then it is generated the *S_o_* output of recognition. This process of calculation is carried out for each module of each level of recognition X_i_ (from X_1_ until X_m_) [33,34].

**Figure 4:**
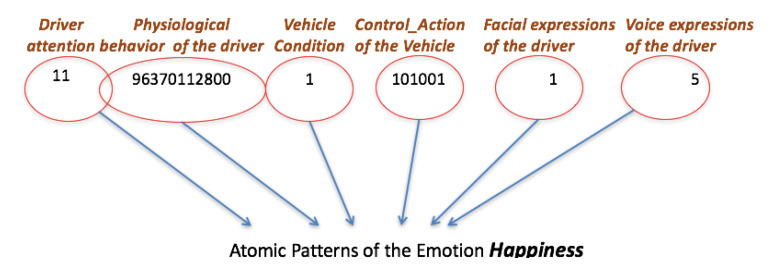
Ar2p model for the Emotion Layer (it recognizes Happiness).

**Figure 5:**
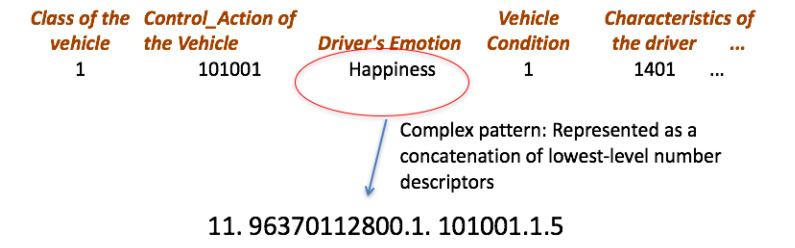
Ar2p model for the State Layer.

Now, it is described how can be modelled the hierarchical pattern using Ar2p. Each level of the hierarchy is defined by a set of descriptors. The application of Ar2p for the driving style recognition is described in detail below. Firstly, the following basic conditions are defined:

- Each descriptor is viewed as a string of numbers that represents it. Normally, all descriptors are considered atomic patterns, i.e., they are not defined by other lower-level (see Figure 4 for the case of the emotions).
- The layers to recognize the driver states and the driving styles are composed of descriptors, and of the emotion descriptor, which is a sub-pattern of lower-level (e.g., see Figure 5 for the case of the driver state). In this example, the emotion pattern is defined by the word “11.9637112800 ...”, which is a pattern with sub-patterns of lower level, as described above.
- The driving styles layer is composed of descriptors of atomic patterns, except the descriptors of the driver states and driver emotions.
- The hierarchical pattern is modelled by three levels, X_1_ for the driver emotions, X_2_ for the driver state, and finally, X_3_ for driving style.

In Figure 4, the descriptors are at the top (first layer), and characterize the conditions of the environment, the vehicle and the driver, considered for the recognition of the emotions. Each descriptor is represented by a code, as is shown in Table 5. The codes define the atomic sub-patterns that constitute the emotion, in this case of happiness.

In Figure 5, the descriptors are at the top and characterize the conditions of the vehicle and the driver, considered for the recognition of the driver state. Unlike the previous figure, each descriptor is represented either by a code (number string) or by a string (complex descriptor). For example, happiness is a complex descriptor that is generated by the recognition of another pattern (see Figure 4).

In this way, according to the hierarchical architecture of Ar2p, the hierarchy of patterns to recognize the driving styles would be as follows: at the X_1_ level is the pattern recognition modules of driver emotions, at the X_2_ level the pattern recognition modules of drive states, and finally, at the X_3_ level the pattern recognition modules for driving styles. An example of a recognition module is shown in Table 7. It represents the recognition of the happiness (X_1_ level, i.e., emotions). Similar tables are defined for the other levels.

Ar2p can deal with uncertain or incomplete knowledge through its recognition axioms. In these cases, Ar2p uses the key signals (descriptors) that characterize the patterns in accordance with the importance weight of each one in the pattern. In this way, during the reasoning, it can recognize a pattern with partial information.

### 4.3. Based on Fuzzy Logic

A fuzzy recognition system is a rule-based fuzzy system composed of a set of inference rules of the type IF <Condition> THEN <Action>, which defines the recognition. Before these rules can be used, all input signals must be converted into fuzzy variables. In general, the basic structure of a fuzzy recognition system consists of three components: a rule base that contains the fuzzy rules; a set of fuzzy variables, each one defined by a set of membership functions; and a reasoning mechanism that performs the recognition procedure [35].

In order to model the hierarchical multimodal model of the driving styles, the multilayer fuzzy classifier system (MFCS) proposed in [36] is used. An MFCS consists of a series of fuzzy systems hierarchically distributed, where the output of a fuzzy classifier system (FCS) is the input of the next FCS. This system has the advantage that the total number of rules of the knowledge base is smaller and simpler than a conventional fuzzy system. That is, the system has the advantage of greatly reducing the number of “if-then” rules, because the conclusions are inferred from the outputs of other FCS.

**Figure 6:**
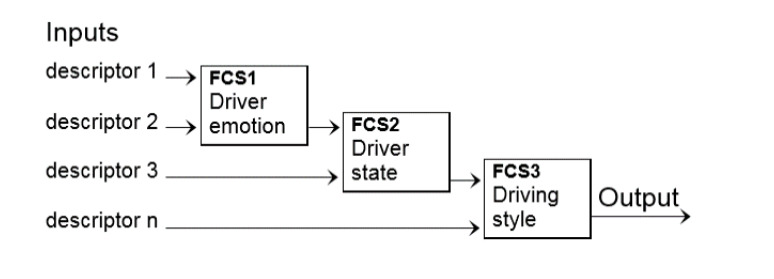
A MFCS model to recognize driving style.

The application of this method for the analysis of driving styles is described in detail below, for its implementation in a real context. In our case, each descriptor is defined as a fuzzy variable, and each level of our hierarchical multimodal model of the driving styles is an FCS. The input of the first FCS are the descriptors of the emotions, and the output is related to the next FCS (driver states) or with the final FCS (driving styles). Figure 6 shows our MFCS model for the recognition of the driving styles, which has three FCSs: (a) the first FCS recognizes the driver emotion, (b) the second FCS recognizes the driver state, and finally, and (c) the last FCS recognizes the driving style.

**Figure 7:**
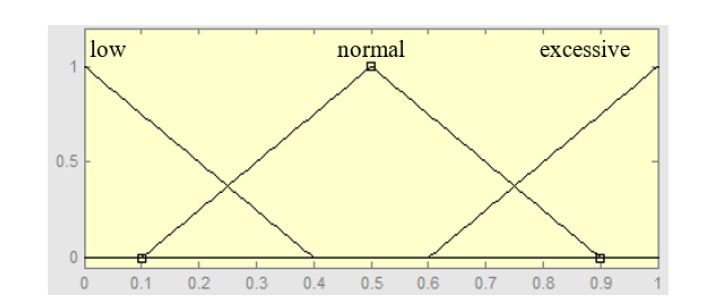
Membership functions of the use-horn fuzzy variable.

The inputs of the FCSs are the same descriptors defined in Section 3 for each level (e.g., road types, weather condition, traffic characteristic, control action on the vehicle, etc.), but, in this case, they are defined as fuzzy variables. Figure 7 shows an example of the membership functions that describe the fuzzy variable about the utilization of the horn. The corresponding fuzzy sets are low, normal, and excessive. This fuzzy variable is input data of the first FCS.

Table 8 shows some examples of fuzzy sets for several descriptors (fuzzy variables) of our hierarchical multimodal model of the driving styles. For example, the use-horn fuzzy variable is defined by three fuzzy sets.

**Figure 8:**
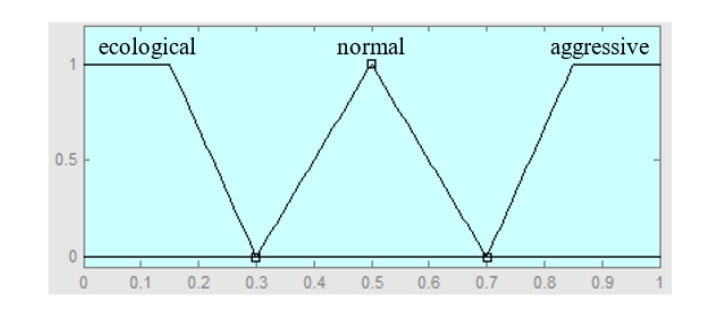
Membership functions of the driving-style fuzzy variable.

Finally, the output of the system is the driving style, which is defined by three fuzzy values, ecological, normal and aggressive, and their membership functions are shown in Figure 8.

The other outputs of the rest of the FCSs are shown in Table 9.

With these fuzzy variables, it is described the set of fuzzy rules of each FCS_i_. Some examples of fuzzy rules to recognize the driver-emotion are:

- If (use-horn is excessive) and (heart rate is high) and (facial expression is grave) then (driver-emotion is anger).
- If (driver hits steering wheel) and (voice is high) and (facial expression is grave) then (driver-emotion is anger).
- If (facial expression is a smile) and (voice is a laugh) and (use-horn is normal) then (driver-emotion is happy).
- Some examples of fuzzy rules to recognize the driver-state are:
- If (driver brakes frequently) and (driver-emotion is anger) and (driver has little experience) then (driver-state stressed).
- If (driver hits steering wheel) and (driver is young) and (driver has little experience) then (driver-state is stressed).
- If (driver-emotion is happy) and (driver is young) and (driver is experienced) then (driver-state is relaxed).
- Finally, some examples of fuzzy rules to recognize the driving-style are:
- If (driver-state is stressed) and (driver-emotion is anger) then (driving-style is aggressive).
- If (driver-state is relaxed) and (weather is raining) and (road has potholes) then (driving-style is normal).
- If (driver-state is relaxed) and (driver-emotion is happy) and (traffic density is free flow) then (driving-style is ecological).

## 5. Comparison of the Approaches

This section presents the comparisons considering the capabilities of each technique at the level of three properties [37]: (i) The reasoning strategies to recognize the driving style (aggressive, normal and ecological), in order to determine the causes of the driving style; (ii) The adaptation strategies to learn the personality of the driver; (iii) The communication of the driving style, which consists into transmit this information to other drivers and that they can understand it.

In the beginning, the experimental database used is described, as well as the metrics used in each property to determine the quality of each approach. Finally, several scenarios are presented for each property.

### 5.1. Experimental Data

**Figure 9:**
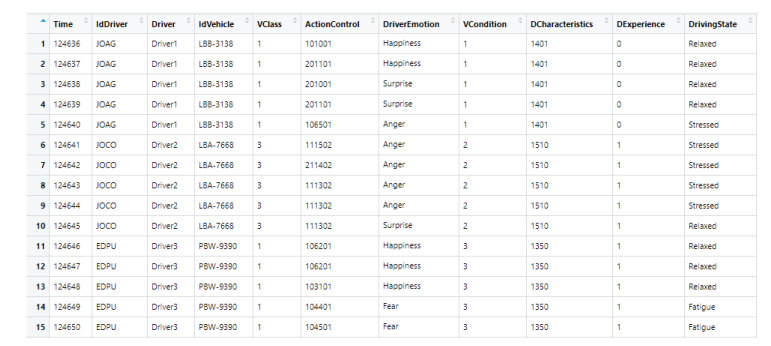
Example of our artificial dataset.

In this section is used an artificial database defined with real data, captured in a multimodal way in different projects that cover the descriptors of each level of our hierarchical model. The merging of different database was necessary, in order to describe driving styles, since there is not a database with the descriptors of our hierarchical data model (see Figure 9). The database combines the set of descriptors based on the time variable. That means, it is supposed the occurrence of a given set of events (values of descriptors) in a given moment, which is registered in the database with this time label. Additionally, it is supposed a specific emotion, driver state, and driving style for each specific moment (register) in the database. It is sufficient for our approaches, in order to learn and to infer the emotions, driver state, and style of driving.

Table 1, Table 2 and Table 3 have defined the set of descriptors for the recognition of a driving style. The descriptors are stored in an artificial database using real data. The data sources of the database are: the “IdDriver”, “IdVehicle” and “Time” variables are generated by us; the “Driver”, “Gender”, “Age”, “Limitation” and “Driving Experience” variables are taken from personal sheets; the “Road_Type”, “Road_Surface_Conditions”, “Special_Conditions_at_Site, and Light_Conditions variables are taken from the Accident_2015 database [38]; the “Pressure”, “Temperature”, and “Wind_Speed” variables are taken from a climate database [39]; the “Density of traffic”, “Type_vehicle”, and “Hands on the wheel” variables are taken from the Waze database [40]; the “Altitude” and “GPS Speed” are taken from GPS devices; and the “Brake_Light”, “Horn use”, “Blood pressure”, “HeartRate”, “BodyTemp” variables are obtained from other sources [41,42].

The variables are combined based on the hierarchical multimodal model for driving style recognition (see Table 1, Table 2 and Table 3) and the key variables defined in Table 10. The relationships between driver emotions, driving styles, and driver state variables are defined by the hierarchical relationship between them defined in our pattern model. In addition to this, the arrangement of the data was chronologically carried out using the key variables (see Table 10) to synchronize the rest of the information, in order to incorporate the descriptors according to the simulated driving event. Initially, the information about the context is included, then the information about the vehicle, and finally, the information about the driver. The consistency is guaranteed because the variation of each descriptor is determined by each source of data, according to the simulated driving style in each specific situation (determined by the key variables).

So, each level within the hierarchical model has all the information for groups of drivers, which was recorded every certain interval of time. The first group of drivers has a short sampling period (15 min) of the descriptors. Another group of drivers has a much larger sample period (several hours), in order to allow changes in all descriptors, especially in weather-related descriptors. Our database has the same structure as Table 5 (it is the conceptual view of the database), but with the key fields defined in Table 10. The keys are the time when taking the sample, and the information about the driver and the vehicle, while the rest of the information corresponds to the values of the descriptors. The database can be downloaded from www.ing.ula.ve/~aguilar/desarrollo-software/VistaMinableOperativa.xlsx.

### 5.2. Metrics

In order to evaluate the recognition systems based on the different paradigms proposed in section four, certain performance criteria are defined, which are grouped into three groups:

- Criteria related to the recognition capability.
- Criteria related to the adaptive ability.
- Criteria related to the ability to communicate the recognized information.

#### 5.2.1. Metrics about Reasoning Capabilities

Consists of detecting anomalous situations, such as negative (aggressive, etc.) driving styles, maybe with additional information about the causes, to inform the ADAS. For that, the metrics about the inference capabilities are important, which allows recognizing and diagnosing. The metrics used in this work are:

Coverage: It verifies the completeness of the technique, i.e., if it represents all possible situations to recognize. Specifically, for the case of the driving style, the proportion of driving styles that cannot be recognized/detected.

(4) $$P_{STYLES}=\frac{1}{\#Styles}\times \overset{i}{\underset{w=1}{\sum }}y_{STYLES_{w}}$$

where $P_{STYLES}$ is the proportion of driving styles that can be recognized, #Styles is the number of driving styles, and $y_{STYLES_{i}}$ is a binary variable that is equal to 1 if the style *i* is recognized, otherwise the value is 0. This expression can be extended to the cases of states and emotions of the drivers. 

Compactness examines the density of the technique, understood by the number of patterns to recognize a state, an object, etc. For the case of the driving styles, it is calculated as the average number of patterns used to recognize the different styles.

(5) $$C_{R_{STYLES}}=\frac{\#Styles}{\#RStyles}$$

where $\;C_{R_{STYLES}}\;$ is the relative compactness of the driving styles, and #RStyles is the number of patterns used during the experimentation to recognize the driving styles. A value close to 1 means more compactness (good value because the techniques can recognize a state, an object, etc. with few information/patterns). This expression can be extended to the cases of states and emotions of the drivers.

Time of reasoning it is the average time to recognize.

(6) $$Time_{R_{t}}=timeEndSimulation_{t}-timeStartSimulation_{t}\;$$

where $Time_{R_{t}}$ is the average time of the technique *t*.

#### 5.2.2. Metrics about Learning Capabilities

This section specifies the metrics about the learning quality of the paradigms.

Precision (*Mp*) determines if the system recognizes the right cases and not others. The result of this operation is between 0 and 1; a perfect precision is 1 when only the correct cases are recognized.

(7) $$Mp=\frac{TREx}{TREx+TRFpx}\;$$

where *TREx* it is the total of successful recognition (true positives), and *TRFpx* is the total of failed responses (false positives).

Recall (*Mr*) is defined as the number of cases that are recognized of the total of cases that must be recognized. If the result of this measure is 1, then it represents a perfect memory, and there is not an informative silence.

(8) $$Mr=\frac{TREx}{TREx+TRFnx}$$

where *TRFnx* is the total of failed answers (that must be recognized) (false negatives).

f-measure (Ma) it measures the general performance of the learning considering recall (*Mr*) and precision (*Mp*).

(9) $$Ma=\frac{2MpMr}{Mp+Mr}$$

Accuracy is the ratio of the number of correct predictions of the total number of inputs.

$$\frac{TREx+TREnx}{TREx+TRFnx+TRFpx+TREnx}$$

where, *TREnx* is the total of successful non-recognition (true negatives).

Quadratic learning error (*EAC*) is the quadratic error between the output that the paradigm gives and the output that should give.

(10) $$EAC=\frac{1}{n}\;\overset{n}{\underset{i=1}{\sum }}{\left(\hat{Sp}-Sp\right)}^{2}$$

where $\hat{Sp}\;$ is a vector of n responses given by the paradigm, and Sp is the vector of answers that it should give.

#### 5.2.3. Metrics about Communication Capabilities

They are oriented to the communication capability of each technique, such that they must transmit faster, and the information transmitted must be understandable by the receptors. In this case, two metrics are used:

Transmission time defines the time to prepare the information to be transmitted with the recognized information by the technique.

(11) $$TT=\frac{real\;transmission\;time}{optimal\;transmission\;time}$$

Processing time defines the time to understand the received descriptors from other sites, in order to be used to recognize a situation.

(12) $$PT=\frac{current\;response\;time}{optimal\;response\;time}$$

### 5.3. Experimental Scenarios

#### 5.3.1. Reasoning Capabilities

This capability consists in the possibility of recognition of different situations and, particularly, the detection of anomalous situations, such as negative driving styles, in order to be useful for the ADAS system, so that it can guide the driver to a positive driving style, which is known to be most suitable for safe driving. The evaluated scenarios are:

- Evaluate the ability to recognize the same situation (emotion, state, or style) through different patterns.
- Study the capacity to recognize the basic emotions, states, or styles in the different drivers.
- Verify the correct functioning of the hierarchical pattern.
- Evaluate the ability to recognize different emotions for the same driver.

In this case, the next metrics are used: coverage, compactness, and time of reasoning. Table 11 shows the average values of these metrics for the scenarios previously defined.

Concerning reasoning capability, chronicles can recognize all possible situations (coverage = 0.98), but it requires a large chronicle database to recognize all possible cases. In this sense, Ar2p is more efficient (compactness = 0.97), since it requires fewer recognition modules by its recursive scheme that reuses information and improves its execution time. In general, the performance of the fuzzy logic is not good, because it needs a large rule database, which does not cover all the possible situations. The same problem can occur with Ar2p, where there are not general patterns for different situations (an advantage of chronicles). Another problem is the reasoning process of the fuzzy logic, which is based on an inference process that can make it very slow at computation time (Time of reasoning = 1.34). In general, a high coverage is desirable, which means the desired situation was successfully recognized. Additionally, a good compactness shows the quantity of information (patterns, descriptors, etc.) to recognize the different situations. Finally, the response time is very important in a real-time context, because an ADAS must quickly assist the driver in a given situation.

The reasoning process of Chronicles based on temporal logic describes naturally the current situation. That is, the reasoning mechanism is based on the events of the descriptors and their temporal relationships, and it manages the incertitude according to when the events occur. In addition, the chronicles define a diagnosis based on the detected causes, to determine the control actions. The only problem is the size of the database of the chronicles, it is required a large database of chronicles to recognize the different situations. Ar2p can reuse much information through the recognition modules, which is an advantage. Also, it can deal with uncertain knowledge. This is achieved within the structures of representation of the pattern (i.e., the pattern recognition modules), with the notion of weight of the descriptors, which support different forms or changes in the descriptors of a pattern. At the level of the reasoning mechanism, it allows inferring a situation, and navigating quickly among the modules. Finally, fuzzy logic allows an approximate reasoning, which implicitly can manage the incertitude, using the idea of imprecision and information granularity in fuzzy descriptors of our multimodal pattern model. The main problem is to obtain the set of rules and the execution time of the MFCS. The MFCS is an excellent strategy to describe the different levels of our pattern model, but it introduces important execution costs in real-time applications.

#### 5.3.2. Learning Capabilities

This capability consists of the ability of adaptation each paradigm for the different situations in the vehicular context, and the personality of the driver. Remember that in some cases, it is necessary the online discovery of new patterns, or the customization of the generic patterns with the specific characteristics of each driver. In this case, the next metrics are used: precision, recall, f-measure, and quadratic learning error. Table 12 shows the average values of these metrics for the scenarios previously defined. We have used a cross-validation approach and added a classic recognition algorithm, random forest (RF), in order to calculate and analyze these metrics in different contexts. In the first one, we have tested our approaches with all the datasets (see Table 12).

Table 12 shows that the chronicles give the best results because they obtain the best precision and recall (like the algorithm Ar2p), but with a minor error. The fuzzy logic normally is based on an elitism procedure based on experts. The FCS allows a learning process, classically based on evolutionary approaches, which is not efficient in real-time situations as the ADAS, while the learning algorithm of Ar2p is quite good, although it converges in a quadratic error superior to the chronicles. RF has similar results that chronicles and Ar2p.

According to the results, the paradigms accurately recognize the patterns, without making other unexpected recognitions. This precision value is because the paradigms learn very specific and unique situations. On the other hand, the good recall indicates that the paradigms can discover all the patterns that a driver experiments during the driving process. In addition, the paradigms recognize the same situation (emotion, state, and/or style) with different patterns, expressing the diversity of context in which the same situation can occur. These results consider the case of online learning and the customization of the patterns, when generic patterns are constructed for each emotion, style, and state (typical, in the case of chronicles and the modules of Ar2p). In general, the quality of the learning algorithm for the chronicles and Ar2p is due to that the learning of patterns is performed whenever a change is detected in the descriptors.

In the second case, 10% of descriptors are randomly deleted during the training phase to prove the capabilities of the methods to learn with partial information. The results are shown in Table 13.

Table 13 shows that the performance of the methods is very similar with respect to the first case, because the methods learn with the available descriptors, and during the testing phase, they can recognize no matter that there are more descriptors captured in the environment (the techniques use the descriptors with which they were trained to recognize new inputs).

In the next test, some descriptors are randomly deleted during the testing phase to prove the capabilities of the methods to recognize with partial descriptors (see Table 14).

In this case (see Table 14), some techniques have difficulties. For example, the fuzzy system in some cases fails to activate the appropriate rules because it is not among the input variables, or the chronicles fail to recognize some situations, despite the fact that the default behaviors defined in them allow partial recognition in some cases. The random forest and Ar2p methods are less affected by these missing data when recognizing, since each tree of RF uses different descriptor combinations and Ar2p the keyword axiom. For a low percentage of missing values, the performance degradation of RF and Ar2p is practically nil.

In the next test, some values are randomly deleted during the testing phase to prove the capabilities of the methods to recognize with partial information (see Table 15).

In general, in this case (see Table 15) the techniques keep more or less their performances. The chronicles have more problems in this case, which can be solved by the actions for default that can be defined in their patterns.

In the last test, with the variables in the dataset for testing are defined data stream, in order to recognize complex driving styles that depend on the sequence of events (see Table 15).

In this case (see Table 16), chronicles work very well because they consider the temporal relationship between the events in their patterns. The fuzzy model keeps more or less its values of quality, because it can include in some cases during the fuzzy inference process (in real-time) the occurrences of new variables. In the case of Ar2p and RF, they cannot represent these types of events (they can recognize specific/current multimodal inputs) and requires the extension of the recognition phase to consider the temporal relationship to be used for these cases.

In general, the chronicles can describe the same situation (an emotion, a driver state, a driving style) using different chronicles. This approach requires a robust chronicle database, which is constantly learned, in order to adapt it to the driver and new situations [30,43]. With respect to Ar2p, it uses two strategies of adaptation [34]. The first one, called new learning, occurs when the input pattern was not recognized (there is not a module that recognizes it). The second one, called reinforcement learning, occurs when the input pattern was recognized. These two learning mechanisms allow a quick adaptation to the driving style of the driver. On the other hand, Ar2p can adapt their pattern recognition modules in accordance with the recognized patterns, readjusting the importance of the weights. Finally, an FCS can learn the rules and the structures of the fuzzy variables. In particular, the membership functions of the fuzzy variables can be adapted to the context, and the rules of the database can be modified (their antecedent and consequent components) [36]. To achieve this, the FCS requires a hard process of modification of the rules, which does not guarantee good results at the level of the learning process (f-measure = 0.80).

#### 5.3.3. Communication Capabilities

In this last case, it is evaluated the capability of each paradigm to transmit the recognized information to other drivers, in a clear way and with semantic meaning. This case is fundamental in the context of IoT, where the exchange of information is between devices, so it must be accurate, contextualized, etc., to be useful [44]. To reach this goal, the transmission of the information must be fast, but additionally, the information sent must be useful for the receptor. In this case, the next metrics are used: transmission time and processing time. Table 17 shows the average values of these metrics for the scenarios previously defined.

In general, the communication times are better for Ar2p, since they simply send a signal, which is recognized by the top-level recognition modules (which can be on different devices). In the case of the chronicles are sent events involved in a recognition process, which must be locally interpreted. These events include the specific information required by the chronicles (such as the emotion experienced by a driver), but it is the only information required. In the case of FCS, the consequent information must be sent, and it is required in the local site a fuzzy reasoning mechanism to process the fuzzy variables, or the fuzzification of the received values. This additional time must be added, to discover the current situation. Thus, the communication in Ar2p are signals between recognition modules, in the chronicles are events, and in FCS fuzzy variables or values that must be fuzzified.

In more detail, in the chronicles, the events can include specific information required by the chronicles. The hierarchical model communicates the events generated by the different descriptors, or the recognition. Ar2p only sends the signals required by the recognition module, which corresponds to a given descriptor (for example, the emotional state of the driver). This signal can be the input of one of the modules of recognition in the other place. Finally, fuzzy logic can send discrete or fuzzy values that must be processed on the other sites, which implies more communication time (Response time = 0.960).

### 5.4. Result Analysis

The data that have been used to build the experimental database are available on the Internet. Our experiments are repeatable using other available data, and they only require the preparation of the data to the format of our database. With respect to our metrics, they allow determining the quality of the results without the necessity of comparing with other works. These metrics evaluate the quality of the different capabilities of the approaches. The metrics as coverage (in the case of reasoning), precision/recall (in the case of learning), and communication time give an idea of the quality of these capabilities.

On the other hand, the patterns and experimental context defined in other work are very different from our study, which makes very complex a comparison with previous works. Nevertheless, we carry out a qualitative comparison with other works. The different methods in the literature about the recognition of the driving style in vehicle drivers, use different recognition methods, descriptors/features, pattern models, and classified driving styles. Table 18 shows a comparison of our method with respect to recent works.

In the literature have been used different techniques as recognition methods, normally based on machine learning. Our paper has added a logical approach as a recognition method, the chronicles. Additionally, the hierarchical pattern model proposed by our work is much more complex, and it includes specific patterns to analyze the driver emotions and the driver states. This work uses the Ar2p pattern recognition technique, which is capable of recognizing patterns with incomplete descriptors, similar to the deep work learning approach used in [20]. Also, this work uses chronicles, which are capable of recognizing complex patterns with temporal relationships between descriptors. Our model considers more descriptors, and can also offer information by level of recognition, which makes it robust and scalable, but [47] can exploit unlabeled data. Also, other works do not recognize the driving style [20,46]. Additionally, our model can be extended to recognize any type of driving style. On the other hand, our hierarchical pattern model is independent of the recognition methods, and inversely, the recognition methods can be used in other pattern models. Finally, this paper analyzes the different approaches with respect to three capabilities very important in the context of autonomous vehicles (IoT): reasoning, learning, and communication.

Furthermore, our methods present very good results for more complex patterns that the existing in the literature. Only the fuzzy logic model has bad results, but for the rest, the metrics of learning and reasoning are very good, close to 1, which speaks of the quality of these paradigms to learn and recognize our pattern of driving style. Finally, some important remarks are: (i) our hierarchical model is more complex than driving style patterns used in the literature, (ii) our hierarchical model includes more classical descriptors, which makes more precise the recognition process (it is the main added value of our pattern model), and (iii) our methods can be applied with partial or ambiguous information about the patterns.

## 6. Conclusions

This paper proposes a hierarchical pattern of driving styles, which considers three levels of recognition, one to recognize the driver emotions, other to recognize the driver states, and finally, the last one corresponds to the driving styles. Our model is flexible because it allows the easy incorporation of new descriptors in the hierarchical model, and it uses the data available in a given moment to recognize. Our model allows incorporating emotional states, driving style, among others, in an ADAS and ACC, to provide greater safety and comfort. The integration in an ADAS is a future work. Particularly, an important contribution of this work is that it covers all aspects necessary to incorporate the human factors in an ADAS, i.e., the ability to recognize driving styles, learn and inform human emotions, among other things. These results can be extrapolated to study the human-machine interaction, within the area known as affective computing. Existing works in the area of recognition in the context of vehicles are usually based on specific descriptors. Another contribution is the theoretical model of patterns proposed, which captures as much information as possible about the driving styles, the driver states, or the driver emotions, making use of a multimodal approach of perception. In this way, it adds a greater amount of information, which makes possible a more precise recognition process.

In addition, the paper analyzes three techniques to recognize the driving style, one based on fuzzy logic, another based on chronicles, and another based on Ar2p. The paper compares these techniques in three cases: their reasoning mechanisms, in order to determine the possible causes or to detect abnormal states; their adaptive capabilities to the drivers; and their communication capabilities of the recognized information, which is very important in the IoT. Each technique has its advantages and disadvantages, and depends on the real context (IoT) to choose one of them. The only technique that does not show good results is the fuzzy model. First, because its learning process is not efficient and is slow. Second, because it requires a large rule base to ensure that all cases are covered. Moreover, and finally, because it needs the definition of messages with sufficient information to understand the information generated by the different sites. On the other hand, the adaptation process in the other approaches (chronicles and Ar2p) allows the discovery of patterns to express the diversity of contexts that may occur during the driving. The ability to reason, in particular, in the chronicles, allows recognizing situations in different ways: the same situation with different patterns, different situations, situations characterized by atomic patterns (e.g., only emotions), or complex situations (described by complex patterns). In the case of communication, there are not problems for Ar2p and chronicles, because the transmissions are signals or events that describe a change of value of a descriptor, or something relevant recognized in a conductor (an emotion, a state or a style).

A future work is to carry out the implementation of these techniques in a real environment, connected to an ADAS. The test cases defined in the current work were developed with an artificial database, using real data. It would be important to test the behavior of the model in real environments with strong time constraints and large amounts of data stream. For such tests, the vehicle must be equipped with systems such as camera, blood pressure sensor, temperature sensor, microphone, GPS, among others, which will allow perceiving in a multimodal way the descriptors that compose the driving pattern. Furthermore, according to the sensors in the real context, the recognition method must be chosen. For example, in a context where perhaps some of the variables may be missing, methods like Ar2p are better, but in a context where high precision based on the temporal relationship of the variables is necessary, the chronicles are better. Then, with the chosen method, the models must be trained, for which it is much better to use methods that have an online learning process (this is the case of Ar2p and the chronicles) to update the patterns over time. Another future work is to test our hierarchical pattern model using support vector machine and deep neural network techniques, in order to compare their performances with the methods proposed in this work.

## Figures and Tables

**Table 1:** Descriptors of the pattern of the driving style.

Descriptor	Description
Type of road	It describes the category of the road. For example, if it is a rural or urban road.
Driver state	It describes the state of the car driver, and it is defined by the second level of our pattern.
Emotion of the driver	It defines the emotional state of the driver, and it is defined by the third level of our pattern.
Weather condition	It characterizes the current weather conditions. For example, rainy, sunny, windy, cloudy, among others.
State of the road	It characterizes the current conditions of the road, the quality of the track. For example, if it is a paved ground, if the road has hollows, etc.
Traffic characteristic	It defines aspects linked to the transit laws, and other road characteristics, in the current context. For example, the speed limits, the traffic signs, among others.

**Table 2:** Descriptors of the pattern of the driving state.

Descriptor	Description
Class of vehicle	It describes the type of vehicle. For example, it can be a car, truck, minivan, etc.
Control Action on the vehicle	It describes the current action of the driver of the car. For example, if the driver is accelerating, braking, etc.
Emotion of the driver	See description in Table 1.
Vehicle condition	It defines the current conditions of the vehicle. For example, if it has a mechanical failure, an electrical failure, the condition of the tires, among other things.
Characteristics of the driver	It defines the profile of age, or physical condition, of the driver. For example, if the driver is a teen, or he/she is an older adult, if the driver has physical limitations, etc.
Driving experience	It characterizes the experience of the driver as a car driver. For example, if the driver has little, medium, or large experience.
Driving hour	It defines the current hour of the day, for example, daytime, night-time hour

**Table 3:** Descriptors of the pattern of the emotions of the driver.

Descriptor	Description
Driver behavior	It defines the current behavior of the driver in the vehicle. For example, the car driver pulls the door, the driver uses the seat belt, etc.
Control Action on the vehicle	See description in Table 2.
Physiological behavior of the driver	It defines the current physiological conditions of the driver. For example, the heart rate of the driver, the blood pressure of the driver, the colour of the face of the driver, etc.
Vehicle condition	See description in Table 2
Voice expressions of the driver	It characterizes the current tone of voice of the car driver. For example, if the driver is shouting, singing, talking normally, etc.
Facial expressions of the driver	It characterizes the current facial expressions of the car driver. For example, if the driver is smiling, he/she is serious, etc.
Body expressions of the driver	It describes the current body expression of the driver. For this, it is necessary the utilization of a body language.

**Table 4:** Driving style [6].

Event Id	Driving Style	Type of Road	Driver State	Emotion of the Driver	Weather Condition	State of the Road	Traffic Characteristic
SD1	aggressive	any	stressed	anger	rainy	the road has potholes	any
SD2	ecological	rural	relaxed	happiness	sunny	any	follows speed limits
SD3	normal	urban	relaxed	happiness	sunny	any	any

**Table 5:** Conceptual view of emotion layer.

Descriptor	Code	Example of the Descriptor
Driver behavior	**XY**	21
	X = represents the gaze (X = 1, look off the road; X = 2, look on the road)	
	Y = represents the hands on the steering wheel (Y = 1, both hands on the steering wheel; Y = 2, only left; Y = 3, only right; Y = 4, none)	
Physiological behavior of the driver	**XXX.XYYY.YZZZ/ZZZW.WWW**	098.0075.1120/0800.001
	X represents the body temperature (normal: 97.7–99.5 °F),	
	Y represents the heart rate (normal: 60–99 bpm),	
	Z represents the blood pressure (systolic/diastolic mmHg),	
	W represents the blood alcohol content (BAC) (% of alcohol for every 100 mL of blood)	
Vehicle condition (e.g., tire condition)	**X**	1
	X represents the condition of the tires (X = 1 new tires (<= 10.000 km of utilization), X = 2 worn tires (between 10.000 and 50.000 km of utilization)	
	X = 3 bad tires (>50.000 km of utilization)	
Control Action on the vehicle	**XYYYYZ**	31001
	X = represents brake light (X = 1, on, X = 2, off, X = 3, any)	
	Y represents GPS Speed	
	Z represents the use-horn (Z = 1 normal; Z = 2 excessive).	
Facial expressions of the driver	**X**	1
	X represents the emotion of the face (X = 1, neutral, X = 2, normal, X = 3, startled, X = 4 serious, X = 5 face with big smiles, X = 6 face with a little smile, X = 7, angry, X = 8, repugnancy)	
Voice expressions of the driver	**X**	2
	X represents the emotion of the voice (X = 1, dry and strong; X = 2, soft and low; X = 3, laugh; X = 4, dry scream; X = 5, neutral).	
Emotion of the driver	**X**	1
	X represents the emotional state of the driver (X = 1, happiness; X = 2, surprise; X = 3, anger, X = 4, fear, X = 5, sadness)	

**Table 6:** Module Structure.

E				
S		C		
Signal ^a^	State	Descriptor(D)	Domain	Weight (w) ^b^
1	False	Descriptor1	<possible descriptor values>	[0, 1]
2	False	Descriptor2	<possible descriptor values>	[0, 1]
3	False	Descriptor3	<possible descriptor values>	[0, 1]
…	…	…	…	[0, 1]
N	False	Descriptor_n_	<possible descriptor values>	[0, 1]
∆*U ^c^*				

^a^ The value of N depends on the pattern to recognize (the descriptors of the pattern). ^b^ All values are normalized [0, 1]. ^c^ Threshold.

**Table 7:** Module of recognition of emotions.

E = Happiness				
S		C		
Signal	State	Descriptor(D)	Domain	Weight (w)
1	F	*Driver_behavior*	<21>	[0, 1]
2	F	*Physiological_behavior_driver*	<96370112800>	[0, 1]
3	F	*Vehicle_condition*	<1>	[0, 1]
4	F	*Control_Action_on_vehicle*	<31001>	[0, 1]
5	F	*Facial_expression_driver*	<1>	[0, 1]
6	F	*Voice_expression_driver*	<2>	[0, 1]
∆*U*				

**Table 8:** Description of some of the input fuzzy variables.

Variable	Fuzzy Sets
Use-horn	low, normal, excessive
Driving experience	little, medium, large
Gaze	eyes off the road, eyes on the road
Hands on the wheel	both, only left, only right, hit the steering wheel
Weather	raining, sunny
Traffic density	flow with restrictions, stable flow, free flow, slow flow, slow flow with stoppage
Facial expressions	neutral, surprise, anger, smile,

**Table 9:** Description of the output fuzzy variables.

Variable	Value
driver-emotion	anger, happy, sad, fear, surprise, neutral
driver-state	relaxed, wakefulness, stressed, pleasant, sleepy, fatigue

**Table 10:** Key variables in the database.

Key	Code	Example of the Key
Time	**XX:YY:ZZ** XX = Represents the hour, YY = Represents the minutes, ZZ = Represents the seconds	12:46:36
IdDriver	Identifier of the Driver	14447345
Driver	Name of the Driver	Juan Perez
IdVehicle	Identifier of the Vehicle	LBB-3138

**Table 11:** Results for the Reasoning Capabilities.

Approaches	Reasoning Capabilities		
	Coverage	Compactness	Time of Reasoning (s)
Fuzzy Logic	0.63	0.65	1.34
Chronicles	0.98	0.73	0.21
Ar2p	0.55	0.97	0.34

**Table 12:** General results for the Learning Capabilities.

Approaches	Learning Capabilities		
	F-Measure	Accuracy	Error
Fuzzy Logic	0.80	0.76	0.69
Chronicles	0.98	0.97	0.02
Ar2p	0.95	0.94	0.10
RF	0.97	0.96	0.08

**Table 13:** Results of the Learning Capabilities with missing data during the training phase.

Approaches	Learning Capabilities		
	F-Measure	Accuracy	Error
Fuzzy Logic	0.80	0.78	0.58
Chronicles	0.97	0.98	0.02
Ar2p	0.96	0.94	0.08
RF	0.97	0.97	0.04

**Table 14:** Results of the Learning Capabilities with missing descriptors during the testing phase.

Approaches	% Missing Descriptors	Learning Capabilities		
		F-Measure	Accuracy	Error
Fuzzy Logic	10	0.75	0.8	0.72
	20	0.64	0.63	0.91
Chronicles	10	0.9	0.89	0.1
	20	0.84	0.84	0.37
Ar2p	10	0.93	0.92	0.1
	20	0.89	0.89	0.16
RF	10	0.94	0.94	0.09
	20	0.92	0.92	0.13

**Table 15:** Results of the Learning Capabilities with missing values during the testing phase.

Approaches	% Missing Values	Learning Capabilities		
		F-Measure	Accuracy	Error
Fuzzy Logic	5	0.80	0.77	0.71
	10	0.72	0.72	0.79
Chronicles	5	0.94	0.93	0.08
	10	0.9	0.89	0.2
Ar2p	5	0.94	0.93	0.1
	10	0.92	0.93	0.16
RF	5	0.93	0.94	0.08
	10	0.92	0.94	0.1

**Table 16:** Results of the Learning Capabilities with missing data during the testing phase with the data stream.

Approaches	Learning Capabilities	
	F-Measure	Accuracy
Fuzzy Logic	0.80	0.79
Chronicles	0.98	0.98
Ar2p	NA	NA
RF	NA	NA

**Table 17:** Results for the Communication Capabilities.

Approaches	Communication Capabilities	
	Response Time	Transmission Time
Fuzzy Logic	0.960	0.770
Chronicles	0.120	0.063
Ar2p	0.093	0.081

**Table 18:** Comparison with other methods.

System	Recognition Method	Pattern Model	Descriptors/Features	Classified Driving Styles
[45]	Statistical-based method: Bayesian probability with kernel density estimation	A single-layer model with the information of all the descriptors	8 features: Acceleration, Yaw rate, Lateral displacement, Vehicle Speed, Steering angle, Physical signal, Physiological signal	Aggressive Normal
[46]	K-means and support vector machine	Two-layer model: one of the physiological signals and other for the driving behavior	physiological signals from electroencephalography (EEG).	Five types of driving behaviors
[19]	Fuzzy logic	Rules-based on the descriptors	Road class, longitudinal acceleration, speed difference, lateral acceleration, Speed difference	Normal comfortable sporty
[20]	Convolutional Neural Network (Deep Learning)	A single-layer model of features defined by the deep learning approach.	Speed norm, acceleration norm, and angular speed, using vehicle sensor data.	Driving patterns: slowdown at hard turns, high-speed driving along straight roads, etc.
[47]	Semi-supervised support vector machine	A single-layer model	Few labeled data points selected from a set of labeled data about the vehicle and context	Aggressive Normal
Our approach	Fuzzy Logic Chronicles Ar2p (Neural Network)	Hierarchical model for the recognition of the driving style.	27 features about the driver, context, vehicle (multimodal descriptors)	Ecological normal aggressive sporty
